# An evidence-based co-occurring disorder intervention in VA homeless programs: outcomes from a hybrid III trial

**DOI:** 10.1186/s12913-018-3123-9

**Published:** 2018-05-05

**Authors:** David A. Smelson, Matthew Chinman, Gordon Hannah, Thomas Byrne, Sharon McCarthy

**Affiliations:** 1 VA National Center on Homelessness among Veterans, Bedford, MA 01730 USA; 2 VA Center for Healthcare Organization and Implementation Research, Bedford, MA 01730 USA; 3 Department of Psychiatry, University of Massachusetts Medical School, 55 N. Lake Avenue, Worcester, MA 01655 USA; 4 VISN 4 Mental Illness Research and Clinical Center, Pittsburgh, PA 15213 USA; 5 RAND Corporation, Santa Monica, CA 90401 USA; 6 Boston University School of Social Work, Boston, MA 02215 USA

**Keywords:** Implementation support, Co-occurring disorders, Fidelity, Training, Technical assistance

## Abstract

**Background:**

Evidence-based treatment for co-occurring disorders is needed within programs that serve homeless Veterans to assist with increasing engagement in care and to prevent future housing loss. A specialized co-occurring disorders treatment engagement intervention called Maintaining Independence and Sobriety Through Systems Integration, Outreach and Networking - Veterans Edition (MISSION-Vet) was implemented within the Housing and Urban Development - Veterans Affairs Supportive Housing (HUD-VASH) Programs with and without an implementation strategy called Getting To Outcomes (GTO). While implementation was modest for the GTO group, no one adopted MISSION in the non-GTO group. This paper reports Veteran level outcome data on treatment engagement and select behavioral health outcomes for Veterans exposed to the MISSION-Vet model compared to Veterans without access to MISSION-Vet.

**Methods:**

This hybrid Type III trial compared 81 Veterans in the GTO group to a similar group of 87 Veterans with mental health and substance use disorders from the caseload of staff in the non-GTO group. Comparisons were made on treatment engagement, negative housing exits, drug and alcohol abuse, inpatient hospitalizations, emergency department visits and income level over time, using mixed-effect or Cox regression models.

**Results:**

Treatment engagement, as measured by the overall number of case manager contacts with Veterans and others (e.g. family members, health providers), was significantly higher among Veterans in the GTO group (B = 2.30, *p* = .04). Supplemental exploratory analyses between Veterans who received “higher” and “lower” intensity MISSION-Vet services in the GTO group failed to show differences in alcohol and drug use, inpatient hospitalization and emergency department use.

**Conclusions:**

Despite modest MISSION-Vet fidelity among staff treating Veterans in the GTO group, differences were found in treatment engagement. However, this study failed to show differences in alcohol use, drug use, mental health hospitalizations and negative housing exits over time among those Veterans receiving higher intensity MISSION-Vet services versus low intensity services. This project suggests that MISSION-Vet could be used in HUD-VASH to increase engagement among Veterans struggling with homelessness, a group often disconnected from care.

**Trial registration:**

Clinicaltrials.gov, registration number: NCT01430741, registered July 26, 2011.

## Background

The Department of Veterans Affairs has undergone a major initiative to end Veteran Homelessness, which included the development and implementation of many new programs and services [1]. One such program is Housing and Urban Development - Veterans Affairs Supportive Housing (HUD-VASH), which is available nationally and combines subsidized housing from Housing and Urban Development and case management services from the Department of Veteran Affairs [2]. While the HUD-VASH Program has reduced homelessness nationally, it has been less effective for Veterans with mental health and substance use [3]. This is of particular concern given that up to 80% of the approximately 48,000 homeless Veterans suffer from mental health and/or substance use disorders [4]. Moreover, co-occurring mental health and substance use problems also often result in treatment discontinuation, housing instability, symptom exacerbations, and use of costly acute treatment services [5]. While a number of treatment approaches have been developed to address mental health and substance use, few have been specifically developed to help homeless Veterans engage in care in an effort to improve behavioral health outcomes and to prevent housing loss, which is a high priority in the Department of Veterans Affairs (VA) [6–8].

Maintaining Independence and Sobriety through Systems Integration, Outreach, and Networking-Veterans Edition (MISSION-Vet) is an evidence-based intervention for co-occurring mental health and substance abuse developed specifically to increase treatment engagement and address the psychosocial needs of homeless Veterans [9–16]. While MISSION-Vet shares some of the similar characteristics as HUD-VASH including a housing first philosophy and assertive community outreach, it also requires certain enhancements like integrated dual disorders treatment, peer support, supported employment and trauma informed care. The MISSION-Vet intervention includes a treatment manual and participant workbook to help providers deliver the intervention, but manuals alone are often insufficient to address institutional barriers and to plan model adaptations that facilitate the implementation of new clinical practices [17]. The presence of such barriers has led to the development and testing of various implementation strategies to improve implementation of integrated behavioral health and substance abuse treatments for those who are dually diagnosed with both mental illnesses and substance abuse disorders [18–21]. However, these studies have yielded modest results, showing that it remains challenging to provide integrated care in typical treatment settings to meet the complex needs for those who are homeless [18–21]. Thus, more research is needed to specifically test the impact implementation strategies have on interventions like MISSION-Vet to improve engagement and other behavioral outcomes in settings that serve those who are dually diagnosed and homeless. Therefore, the primary aim of the current paper was to examine treatment engagement among Veterans served by case managers delivering MISSION-Vet to Veterans served by case managers whom did not get MISSION-Vet. Secondary aims were to assess whether MISSION-Vet increased time to housing loss, reduced hospitalizations and emergency department visits and improved mental health and substance abuse problems.

## Methods

**Fig. 1:**
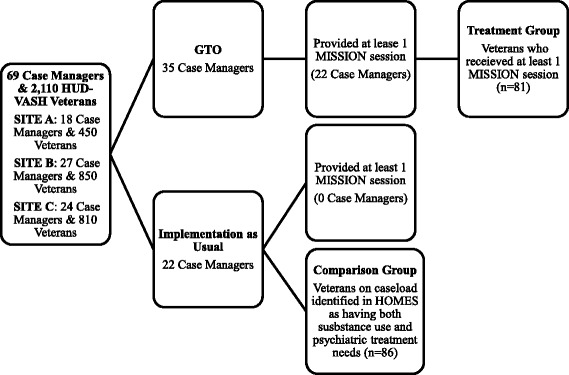
Enrollment Flow Chart. Note: 12 Case Managers overall refused to participate

The VA’s Quality Enhancement Research Initiative funded a Hybrid type III implementation and effectiveness trial of MISSION-Vet within HUD-VASH [22]. This trial used Getting To Outcomes, an implementation support approach, to help treatment teams within HUD-VASH adopt and implement MISSION-Vet [16]. As shown in Fig. 1, this Hybrid Type III trial was carried out in three large VA HUD-VASH programs: Site A (450 Veterans in HUD-VASH receiving housing support and 18 case managers), Site B (850 Veterans in HUD-VASH receiving housing support and 27 case managers) and Site C (810 Veterans HUD-VASH receiving housing support and 24 case managers), for a total of 2110 HUD-VASH Veterans and 69 case managers [16, 23]. At each site, each team was comprised of two sub-teams of case managers, who were randomized (along with the Veterans they serve) to Implementation as Usual (IU) or GTO by the project statistician using a random number generator (see Ref [23] for more details on the methods). In both groups, HUD-VASH staff were instructed on how to engage Veterans into MISSION-Vet services and follow the inclusion and exclusion criteria of: (1) a current substance abuse or dependence disorder and a co-occurring mental illness; (2) is willing to participate in MISSION-Vet services. All 69 HUD-VASH case managers at these sites were invited to a MISSION-Vet Training Webinar. Among those 69, 12 refused to participate, but did not offer reasons for declining. Among those whom elected to participate, 22 were in the IU group and 35 were in the GTO implementation support group across the three sites. While both conditions also received standard MISSION-Vet manuals, the GTO group received additional materials, training, technical assistance, and data feedback (see below).

A recent paper compared the implementation of MISSION-Vet between the GTO and IU groups. Sixty-eight percent of the case managers in the GTO group adopted MISSION-Vet whereas none adopted it in the IU group; implementation uptake was modest with 70% of Veterans with case managers in the GTO group receiving at least one MISSION-Vet session but none receiving a full dose of the intervention [23]. Among the 35 case managers across the three sites enrolled in the MISSION-Vet with GTO group, 22 case managers provided at least one or more MISSION-Vet session to a total of 81 Veterans eligible for MISSION-Vet services on their caseloads. While the original hybrid type III design planned to compare Veterans who received MISSION-Vet in IU and GTO groups [16], no IU case managers provided a single MISSION-Vet session to any Veterans on their caseload whom met eligibility criteria. Thus, after the sites’ GTO sub-team clinicians had concluded delivering MISSION-Vet, we used Homeless Management Evaluation System (HOMES), a data management tool within the Veterans Health Administration that provides longitudinal information about the status of Veterans who have experienced homelessness or are at risk to identify an alternative comparison group. This group was made from among the 567 Veterans on the caseload of the 22 case manager/peer teams in the IU group. Specifically, we identified for inclusion in the comparison group all the Veterans who have co-occurring mental health and substance abuse problems based on their most recent HOMES Assessment (*n* = 87).

The study was approved by the VA Central IRB and case managers consented to participate. The study received a waiver of consent to use the client-level data from the VA’s existing HOMES database to assess MISSION-Vet outcomes.

### Treatment and implementation approaches

The MISSION-Vet treatment intervention is listed in the Substance Abuse and Mental Health Services Registry of Evidence Based Practices [9]. MISSION-Vet is a comprehensive co-occurring disorders intervention with the primary goal of increasing treatment engagement [16]. The 3 core treatment components utilized in MISSION-Vet include Critical Time Intervention (CTI) designed to remove situational barriers to attending treatment [24], integrated co-occurring treatment using Dual Recovery Therapy (DRT) [25] designed to increase motivation to address mental health and substance use, and Peer Support delivered by peer specialists (individuals in recovery from mental illness and substance abuse disorders, trained to use their recovery experience to help others with similar problems engage in treatment) [26]. MISSION-Vet also includes Vocational/Educational Support [27] to help clients obtain and maintain employment and education. Finally, MISSION-Vet includes Trauma Informed care [28] which is designed to help clients address any trauma issues, including making referrals to more structured Post Traumatic Stress Disorders Treatment if needed. MISSION-Vet has a structured treatment curriculum that is outlined in the MISSION implementation materials, which include Treatment Manual and accompanying Consumer Workbook. Moreover, these materials serve as how-to-guides that describe model components, suggestions for service delivery, and provide self-help materials for the client to reinforce skills being taught by the treatment staff [12, 13]. Optimally, each client receives approximately 2.5 h of services a week from a case manager/peer specialist team with treatment intensity reduced over time. This includes 13 structured DRT groups, 11 structured peer specialist groups, and 40 unstructured linkage sessions delivered by a case manager and a peer specialist for a total of 64 sessions over 12 months of treatment. At each site, both sub-teams received a webinar by the MISSION-Vet developer on the model, how to deliver the services and how to utilize the treatment manual and participant workbook [12, 13].

GTO is an implementation support approach used to strengthen the knowledge, attitudes and skills case managers and peer specialists need to carry out MISSION-Vet. GTO involves guiding practitioners to complete steps in three general areas: (1) planning – e.g., developing goals and performance targets, ensuring staff are trained in the evidence-based program; (2) implementation – e.g., monitoring progress, maintaining adherence to an evidence-based program model, and (3) evaluation – e.g., tracking patient outcomes, using data to improve program operations [16, 23]. Each sub-team assigned to GTO assembled a “GTO Planning Team” of staff, led by a designated point of contact. Each sub-team was supported by the GTO Technical Assistance (TA) staff person (SM, in Pittsburgh) who guided the GTO Planning Team through the GTO process, using four key supports: (1) Manual of tools (the manual *Getting To Outcomes in services for homeless Veterans: 10 steps for achieving accountability* [29]. (2) Training. Each sub-team received a six-hour training on using GTO to plan, implement, evaluate, and conduct quality improvement on MISSION-Vet. (3) GTO TA. GTO TA is similar to “facilitation” in the implementation science literature [30]. The GTO TA staff person met with each sub-team about every other a week for about for 18–23 months. (4) MISSION-Vet service tracking—MISSION-Vet service data was collected with a Computerized Patient Record System (CPRS) note template we developed for each team and was fed back to stimulate quality improvement discussions.

### Measures

We examined the following set of outcome measures: 1) Treatment engagement (number of face-to-face contacts, number of contacts of any kind with Veteran, overall number of contacts with Veteran and others [e.g. family members, health care providers, community agencies]); 2) Drug and alcohol use, which was a dichotomous measure based on case managers’ clinical assessment of Veterans’ drug or alcohol use as either dependence or severe dependence; 3) Any inpatient hospitalization for medical and mental health conditions; 4) Any emergency department visit for medical and mental health conditions; and 5) negative housing exits from HUD-VASH. The first four of these outcomes (i.e. treatment engagement, drug/alcohol dependence, inpatient hospitalizations, emergency department visits) measures were assessed monthly for 12 months following study enrollment. These measures were obtained from the standardized monthly and quarterly status reports completed by case managers, which were extracted from HOMES. We obtained information on negative housing exits from the HUD-VASH Exit form, which is available in HOMES and tracks the dates and reasons for all exits from the program. We measured whether and when a Veteran experienced a negative housing exit over the period from study enrollment (this was the first date of MISSION-Vet for those served by case managers in the GTO group and the date of the first MISSION-Vet session provided to any Veteran at the appropriate study site for those in the comparison group) until the end of the study observation period. The maximum follow-up time for capturing exits was 2.4 years, with variable follow-up times due to different dates (in calendar time) of entry into the study and corresponding differences in the availability of follow-up data on housing outcomes from HOMES. A negative exit from HUD-VASH was defined as one that occurred due to non-compliance with case management, eviction, Veteran dissatisfaction with housing, inability to locate Veteran and incarceration.

### Data analyses

We compared changes in the first four outcomes (Treatment engagement, drug/alcohol dependence, inpatient hospitalizations, emergency department visits) among Veterans served by case managers in the GTO group relative to the comparison group of Veterans, by fitting a series of mixed-effects regression models to account for clustering of observations within individuals and case managers, using linear models and SAS PROC MIXED for continuous outcomes and binary logistic models and SAS PROC GLIMMIX for dichotomous outcomes. Final mixed-effect models for all outcomes included fixed terms for group (GTO group vs. comparison), time, group x time interaction as well as control covariates for age, year of move-in to HUD-VASH, and the following measures obtained from a VA homeless program intake form: need for treatment for a medical problem; employment status (unemployed, employed, not in labor force); and housing status at time of assessment (stably housed, unstably housed, or unknown). We used Kaplan-Meier survival curves to estimate the extent and timing of negative housing exits over time and Cox proportional hazards regression to assess the relationship between membership in the GTO group and risk of experiencing a negative housing exit, adjusting for a set of relevant covariates.

Inspection of data collected on MISSION-Vet fidelity suggested low to moderate fidelity, with 20% of Veterans served by case managers in the GTO condition receiving only 1 or 2 MISSION-Vet Sessions, two thirds receiving 19 or fewer of the proposed 64 sessions and none receiving the full intervention. As such, we conducted a supplemental exploratory set of analyses to assess the relationship between intensity of MISSION-Vet and the outcomes of interest in which we: 1) treated the number of MISSION-Vet sessions received per month as a continuous variable, and 2) stratified Veterans in the GTO case manager group into “higher” and “lower” intensity groups, using the median number of MISSION-Vet sessions received (11 sessions) as the delimiting factor.

## Results

Bivariate tests found that, relative to the comparison group, Veterans served by the GTO group were significantly older (55.9 years vs. 49.7 years, *p* < .001), had been in HUD-VASH housing longer (57.1% in housing for 3 years or more vs. 42.1% in housing for 3 years or more, *p* < .01), were less likely to need treatment for a medical problem (63.2% vs. 82.6%, p < .001), but were no different in terms of employment (13.1% employed vs. 13.8% employed, *p* = .76) and housing status at time of assessment (78.6% homeless vs. 68.1% homeless, *p* = .58).

As shown in Table 1, the mixed-effects models found that all three measures of treatment engagement were significantly higher, on average, across time among Veterans in the GTO group relative to the comparison group. When considering the overall number of case manager contacts with Veterans and others (e.g. family members, health providers), Veterans in the GTO group had, on average, more contacts with case managers during the study period (B = 2.30, *p* = .04). However, these measures of treatment engagement declined significantly over time for both groups, and the negative time by GTO interaction term (B = − 0.19, *p* = .01) indicates that the difference in treatment engagement between Veterans in the GTO group and those in the comparison group attenuated over time. Of note, this gradual reduction in service intensity in the GTO group is consistent with the MISSION-Vet service delivery schedule, whereas the comparison group had consistently lower intensity throughout the observation period. Neither the main effect of membership in the MISSION GTO group, nor the interaction with time were statistically significant predictors in the other outcome models, as shown in Tables 2 and 3. This pattern was similar in the analyses that used continuous and categorical MISSION-Vet predictors.

**Table 1:** Results of Mixed Effect Models for Engagement with Case Management Services (*n* = 168)

	All Contacts		Face to Face Contacts		Case Manager Contacts	
	B	*p*	B	*p*	B	*p*-value
Main Analysis (GTO vs. Comparison group)						
Time	−0.12	0.04	− 0.05	0.02	− 0.08	0.03
GTO group^a^	2.32	0.04	0.93	0.02	1.49	0.02
Time x GTO group	−0.19	0.02	−0.06	0.08	−0.12	0.03
Supplemental Analysis (Categorical MISSION Intensity)						
Time	−0.27	<.01	−0.14	<.01	−0.2	<.01
Comparison group^b^	2.54	0.06	2.08	<.01	2.7	<.01
High Intensity MISSION group^b^	1.09	0.46	1.64	<.01	1.47	0.06
Time x Comparison group	−0.13	0.18	−0.1	<.01	−0.14	<.01
Time x High Intensity MISSION group	0.24	0.09	0.01	0.80	0.14	0.10
Supplemental Analysis (Continuous MISSION Intensity)						
Time	−0.17	<.01	−0.05	0.01	−0.09	<.01
# of MISSION Sessions	0.89	<.01	0.67	<.01	0.78	<.01
Time x # of MISSION Sessions	−0.03	0.19	−0.03	<.01	−0.03	0.04

Models are linear mixed effects models assessing change in outcomes over each month during the 12-month period following study enrollment. All models are adjusted for Veteran age, year of move-in to HUD-VASH, Veteran need for treatment for a medical problem; employment status and housing status at time of assessment

^a^vs. Comparison group

^b^vs. Low intensity MISSION group

**Table 2:** Results of Mixed Effect Models for Drug and Alcohol Use (*n* = 168)

	Drug Use		Alcohol Use	
	AOR^a^	*p*	AOR^a^	*p*
Main Analysis (GTO vs. Comparison group)				
Time	1.15	0.23	1.13	0.18
GTO group^b^	0.27	0.41	0.05	0.03
Time x GTO group	0.84	0.19	0.83	0.11
Supplemental Analysis (MISSION Intensity)				
Time	1.06	0.35	1.06	0.28
Comparison group^c^	2.83	0.27	1.73	0.47
High Intensity MISSION group^c^	0.18	0.07	0.33	0.21
Time x Comparison group	1.04	0.64	1.07	0.33
Time x High Intensity MISSION group	1.04	0.66	1.01	0.92
Supplemental Analysis (Continuous MISSION Intensity)				
Time	0.94	0.58	0.95	0.51
# of MISSION Sessions	0.73	0.44	0.72	0.13
Time x # of MISSION Sessions	1.08	0.09	1.05	0.13

Models are linear mixed effects models assessing change in outcomes over each month during the 12-month period following study enrollment. All models are adjusted for Veteran age, year of move-in to HUD-VASH, Veteran need for treatment for a medical problem; employment status and housing status at time of assessment except for supplemental analysis model of alcohol use, which is not adjusted for any covariates due to convergence failure when including covariates

^a^Adjusted odds ratio

^b^vs. Comparison group

^c^vs. Low intensity MISSION group

**Table 3:** Results of Mixed Effect Models for Emergency Department and Inpatient Hospitalization

	Emergency Department				Inpatient Hospitalization			
	Medical		Mental Health		Medical		Mental Health	
	AOR^a^	*P*	AOR^a^	*P*	AOR^a^	*p*	AOR^a^	*p*
Main Analysis (GTO vs. Comparison group)								
Time	1.01	0.95	1.14	0.51	0.97	0.86	1.38	<.01
GTO group^b^	2.96	0.19	2.6	0.48	0.69	0.73	1.07	0.97
Time x GTO group	0.86	0.18	0.84	0.29	1.03	0.85	0.88	0.333
Supplemental Analysis (MISSION Intensity)								
Time	0.95	0.55	1.12	0.63	0.95	0.77	4.34	0.3
Comparison group^c^	0.63	0.53	0.37	0.55	5.2	0.22	0.04	0.13
High Intensity MISSION group^c^	3.06	0.24	1.46	0.87	6.56	0.19	0.05	0.14
Time x Comparison group	1.07	0.5	1.12	0.54	0.97	0.85	11.11	0.1
Time x High Intensity MISSION group	0.91	0.58	0.75	0.34	1.03	0.89	11.11	0.13
Supplemental Analysis (Continuous MISSION Intensity)								
Time	0.9	0.35	1.05	0.82	0.98	0.89	0.87	0.45
# of MISSION Sessions	1.12	0.56	0.68	0.41	1.35	0.17	0.51	0.28
Time x # of MISSION Sessions	1.02	0.54	1.06	0.17	1.00	0.93	1.07	0.3

All models are adjusted for Veteran age, year of move-in to HUD-VASH, Veteran need for treatment for a medical problem; employment status and housing status at time of assessment except for supplemental analysis models of mental health emergency department visits and medical inpatient hospitalizations, which were not adjusted for any covariates due to convergence failure when including covariates

^a^Adjusted odds ratio

^b^vs. Comparison group

^c^vs. Low intensity MISSION group

With the categorical predictor, those in the GTO group who met the criteria for higher intensity MISSION-Vet had significantly more face to face case manager contacts than those in the lower intensity MISSION-Vet group and the magnitude of this relationship did not change over time. With respect to the continuous MISSION-Vet predictor, the number of MISSION-Vet sessions was positively associated with all three case manager contact measures, although the strength of this relationship attenuated over time for face to face and case manager contacts. There were no significant relationships between the categorical and continuous MISSION-Vet measures (or their interaction with time) and the other outcome measures, although in both cases there were consistently non-significant negative relationships between receipt of MISSION-Vet services and the probability of alcohol use, drug use, and mental health hospitalizations.

The Kaplan-Meier estimates found that 11.6% of Veterans in the GTO group and 19.2% of Veterans in the comparison group had experienced a negative housing exit at 2 years following study enrollment. However, the Cox regression model found no significant association between group membership and risk of negative housing exit (HR = 1.43, *p* = .481).

## Discussion

This study assessed whether MISSION-Vet, as practiced in real-world clinical settings, improved Veteran outcomes. Given that none of the case managers in IU adopted MISSION-Vet, Veterans served by those case managers who met criteria for mental health and substance use problems were used to form a comparison group. The most salient finding was that treatment engagement was significantly higher across time among Veterans in the GTO group relative to the comparison group. This finding occurred despite modest fidelity to the MISSION-Vet services in the GTO group. Among homeless individuals with co-occurring mental health and substance use in Permanent Supportive Housing, treatment engagement is an important first step in the recovery process, but individuals might need more time and longer term supports to address mental health and substance use [31]. Furthermore, others have also documented successful treatment engagement through the use of multifactorial treatment interventions like MISSION-Vet as well as the inclusion of the evidence based critical time intervention approach [32, 33].

It is noteworthy that while Chinman et al. [23] previously documented that case managers faced significant implementation challenges, even a modest amount of MISSION-Vet increased treatment engagement compared to Veterans served by case managers whom did not offer MISSION-Vet. Despite the finding on increasing treatment engagement among those receiving MISSION-Vet, this study failed to find statistically significant improvements in hospitalizations, mental health, or substance abuse outcomes that were found in other MISSION-Vet clinical trials [14, 15]. These results are not surprising given lower than expected MISSION-Vet services being delivered. Moreover, no Veteran treated by staff in the GTO group received a full “dose” of MISSION-Vet. Furthermore, a secondary analyses of service intensity suggested that while not statistically significant, clients receiving more MISSION-Vet services tended to have negative relationships with the probability of alcohol use, drug use, and mental health hospitalizations over time compared to clients who received lower intensity MISSION-Vet services. However, this finding may be partially due to the existence of relatively more intensive needs among the low-intensity MISSION-Vet group, resulting in an increased difficulty keeping clients continuously engaged in MISSION-Vet services and also explain the relatively less desirable set of observed outcomes.

Despite the modest findings and lessons learned from this study, it is important to acknowledge several limitations. First, this study included a small sample and a comparison group that was not perfectly matched. Second, the treatment group only delivered a modest amount of the MISSION-Vet services as compared to the prescribed protocol of services. Third, the outcome measures were made up of medical record extraction of clinician ratings as opposed to the use of primary data collection of client improvement.

## Conclusions

Despite the modest amount of MISSION-Vet being delivered, this data suggests that the intervention could assist with increasing service intensity among a group that is often disengaged from care. This might be useful for reducing HUD-VASH negative exits, an outcome that the Department of Veterans Affairs is particularly interested in addressing. Moreover, while not statistically significant, perhaps partially due to the modest sample size, those in MISSION-Vet had about half the number of negative HUD-VASH exits compared to the comparison group (11.7% versus 19.2%). With regards to future directions, we recognize that MISSION-Vet is a complex intervention that includes the delivery of five evidence-based practices, which could have made it more difficult for HUD-VASH case managers to fully implement. Future research might systematically vary the MISSION-Vet components in a disaggregate study design to assess the relative contribution of each component. This would ultimately help to examine whether delivering only a portion of the MISSION-Vet services would demonstrate a robust effect as was the case on our prior trials with clients receiving the full dose of the MISSION-Vet services.

## Abbreviations

AOR: Adjusted odds ratio
CPRS: Computerized patient record system
CTI: Critical time intervention
DRT: Dual recovery therapy
GTO: Getting to outcomes
HOMES: Homeless management evaluation system
HUD-VASH: Housing and urban development-veteran affairs supportive housing
IU: Implementation as usual
MISSION-Vet: Maintaining independence and sobriety through systems integration, outreach and networking- veterans edition
TA: Technical staff
VA: Veterans affairs
